# Smoothed particle hydrodynamics simulation of a laser pulse impact onto a liquid metal droplet

**DOI:** 10.1371/journal.pone.0204125

**Published:** 2018-09-25

**Authors:** Phoevos Koukouvinis, Nikolaos Kyriazis, Manolis Gavaises

**Affiliations:** School of Mathematics, Computer Science & Engineering, Department of Mechanical Engineering & Aeronautics, City University of London, Northampton Square, London, United Kingdom; Heidelberg University, GERMANY

## Abstract

The impact of a laser pulse onto a liquid metal droplet is numerically investigated by utilising a weakly compressible single phase model; the thermodynamic closure is achieved by the Tait equation of state (EoS) for the liquid metal. The smoothed particle hydrodynamics (SPH) method, which has been employed in the arbitrary Lagrangian Eulerian (ALE) framework, offers numerical efficiency, compared to grid related discretization methods. The latter would require modelling not only of the liquid metal phase, but also of the vacuum, which would necessitate special numerical schemes, suitable for high density ratios. In addition, SPH-ALE allows for the easy deformation handling of the droplet, compared to interface tracking methods where strong mesh deformation and most likely degenerate cells occur. Then, the laser-induced deformation of the droplet is simulated and cavitation formation is predicted. The ablation pattern due to the emitted shock wave and the two low pressure lobes created in the middle of the droplet because of the rarefaction waves are demonstrated. The liquid metal droplet is subject to material rupture, when the shock wave, the rarefaction wave and the free surface interact. Similar patterns regarding the wave dynamics and the hollow structure have been also noticed in prior experimental studies.

## 1 Introduction

The continuation of Moore’s law [1], in order to meet up all the increasing demand in power efficiency, has been the main driver for the development of photolithography, which is a microfabrication process that patterns parts of a thin film aiming to make printed circuit boards. Applications of laser-induced deformation of a liquid metal droplet that this study deals with, can be found in photolithography, so as to shrink chip features, aiming to pack more and more transistors on a single chip.

Moore’s law indicates that the number of the transistors and other electronic components per integrated circuit should double every two years, increasing that way the computational power. However, there are some natural limitations that have to be surmounted in order to achieve faster processing, since currently chip features have already reached nanometer-size dimensions [2, 3]. The minimum wavelength that is achieved and it is widely used is in deep Ultra Violet (DUV) lithography at λ = 193 *nm*; this is practically the smallest linewidth that can be imaged. Further downgrading the chip features requires advances in the lithography field. The most promising method is the extreme Ultra Violet (EUV) lithography [2, 4, 5] at an illumination wavelength of λ = 13.5 *nm*, which is actually one order of magnitude smaller than DUV. In comparison to DUV lithography, where the wafer is surrounded by air, in the EUV lithography the laser operates in a large vacuum chamber. Another difference is that in EUV the liquid metal droplets are deformed due to two stages of laser impact [6–8].

Although several studies regarding droplet impacts, either theoretical, experimental or numerical have been published (such as [9–12]), only a few have focused on droplet fragmentation [13–15] or on liquid metal droplets. The lack of experiments on laser-induced shocks for liquid metals [16–18] lies in the fact that the operated temperature must be significantly higher compared to the corresponding experiments for water [15, 19–22]. Another difference in liquid metal droplets is the intensity of the shock waves, which is of Mbar-scale and as a result, the droplet is strongly deformed (ablation) and a hollow structure consisting of two lobes is formed in the middle [23]. Similar findings have been published by Vinokhodov et al. [24], who studied the dynamics of liquid metal droplets under ultra-short laser pulses, aiming to optimise the shape of the droplet target used in EUV. They identified two different expanding shells and a waist between, 1 *μs* after the pulse was emitted, where the right shell was expanding faster than the left one until it reached a critical thickness and then it burst. Basko et al. [25], employed experimental, theoretical and numerical methods for studying the deformation and fragmentation of a metal droplet by sub-picosecond and picosecond laser pulses. Recently, Krivokorytov et al. [26] impacted metal droplets with sub-picosecond laser pulses and they captured their expansion and fragmentation, as well as high-speed jets starting from the droplet surface. The cause of this jetting phenomenon is cavitation formation at the center of the droplet, due to the rarefaction wave and the number of the jets was correlated to the laser energy by coupling the cavitation bubble dynamics with Rayleigh-Taylor instabilities.

The driving mechanism in laser pulse impacts onto water or liquid metal droplets is the ablation pressure [27–29] and its impact on the droplet shape is demonstrated in the following studies. Klein et al. [30] showed the propulsion, deformation and fragment of a water droplet, when laser energy was deposited on it. In a follow up work, Gelderblom et al. [31] studied a free falling water drop hit by a nanosecond laser pulse and investigated the effect of the pulse shape and the droplet properties on its deformation. More recently, Reijers et al. [8] modelled the droplet deformation for short ablation pressure duration. They found that for small transfer of momentum the shape of the droplet resembles of an incompressible droplet deformation. However, for high momentum transfer, the droplet shape is strongly affected by the pulse duration and the compressed areas significantly modify the droplet shape.

Since the utilised numerical approach in the present study is the SPH-ALE method, a short literature review is given. SPH method was originally developed for astrophysical problems in 1977 by Lucy, Gingold and Monaghan [32, 33], however 15 years later, SPH was extended to free surface flows by Monaghan [34] and has been widely used for interfacial flows [35, 36]. One application of SPH method is in ocean engineering and it has been employed by Dalrymple and Rogers [37] for modelling water waves and later on, for wave-body interaction [38]. Furthermore, Marrone et al. [39] added a diffusion flux in the SPH method and solved impact flow problems (see also [40]). Vila [41] introduced the mathematical framework of the SPH-ALE, so as to overcome the drawbacks of the standard SPH method. In a follow-up study, Marongiu et al. [42–45] applied the method in the free surface flow of Pelton turbines and later on, they implemented the method for GPU processors [46, 47]. In an effort to provide solution for unsteady FSI problems, Li et al. [48] coupled the SPH-ALE method with a Finite Element structural solver. Time advancement was achieved by the Newmark scheme for the solid part and by a 2nd order in space Runge-Kutta method for the liquid part.

While several experiments of liquid metal droplet deformation due to a laser pulse have been performed, the only similar numerical work is of Reijers et al. [8, 49], where they employed an axisymmetric Lattice-Boltzmann method to compare with the derived single-phase analytical pressure field. In the present study, the SPH method within the ALE framework has been utilised in order to model the waves travelling inside the liquid metal droplet and to identify the two cavitation regimes. To authors’ best knowledge, this is the first effort trying to give an insight on the impact of cavitation in such phenomena within the SPH-ALE framework. The suggested methodology is ideal and numerically efficient for free surface flows. Traditionally, SPH method can easily handle material deformation, without the need of mesh deformation techniques, such as in Lagrangian-type methods (see [9, 50]) or an explicit interface treatment, for instance in VOF-like methods. The boundary conditions are simply enforced here, which is not so straightforward in front-tracking methodologies. Furthermore, advection-type methodologies are prone to numerical diffusion due to the Eulerian approach, either if the gas phase is modelled [51], or if the gas phase is omitted [52].

Regarding the assumptions of the present simulation, the gas phase is not modelled here due to the vacuum conditions surrounding the metal droplet. In addition, phase-change is not modelled here since the density is infinitesimal in the cavitation regime due to the very low saturation pressure. Surface tension is neglected in the present simulation (the Weber number is *We* > 4 ⋅ 10^6^), as it doesn’t affect the violent dynamics of bubble nucleation (shock focusing, rarefaction wave and liquid tension) as it can be seen in S3 Appendix, which is the main focus of the present study. However, surface tension will play an important role at later times of the bubble and droplet evolution that are not modelled in the present work.

The paper is organized as follows. In section 2 the SPH-ALE method is briefly described. In section 3 the results of the deformed droplet are presented and discussed and the most important conclusions are summarised in section 4. Validation of the numerical method is presented in S1 and S2 Appendices, whereas in S3 Appendix, the effect of the surface tension and the viscosity are investigated.

## 2 Numerical method

The numerical method is based on Vila [41], Marongiu and Parkinson [42] and it has been developed and validated in [53, 54] for several cases, such as implosions, explosions, impacts, impingements and hydrodynamics.

In the present study, the flow is considered inviscid and thus, viscosity terms are omitted (see S3 Appendix). Furthermore, the fluid is assumed to be weakly compressible, given the fact that the speed of sound in the liquid is ∼ 2000 *m*/*s* and in the air ∼ 300 *m*/*s*, and the Tait equation of state has been used for calculating the pressure field from the density field, avoiding this way to solve the Poisson equation.

### 2.1 SPH-ALE formulation

The Euler equations using the ALE description were introduced by Vila [41]:

$$L_{u0}\left(\Phi \right)+\frac{\partial }{\partial x^{i}}(F_{E}^{i}-u_{0}^{i}\Phi )=0,$$ (1)

where $L_{u0}\left(\Phi \right)=\frac{\partial \Phi }{\partial t}+\frac{\partial (u_{0}\Phi )}{\partial x}+\frac{\partial (u_{0}\Phi )}{\partial y}+\frac{\partial (u_{0}\Phi )}{\partial z},$

**Φ** is the conservative solution vector: $\Phi =[\begin{array}{c} \rho \;\rho u\;\rho v\;\rho w \end{array}{]}^{T}$

$u_{0}^{i}$ is the i-component of the transport field velocity vector: **u**_0_ = (*u*_0_, *v*_0_, *w*_0_) and **F**_*E*_ is the 3D inviscid flux tensor:

$$\begin{array}{c} F_{E}^{1}=[\begin{array}{c} \rho u\\ \rho u^{2}+p\\ \rho uv\\ \rho uw \end{array}],\;F_{E}^{2}=[\begin{array}{c} \rho v\\ \rho vu\\ \rho v^{2}+p\\ \rho vw \end{array}],\;F_{E}^{3}=[\begin{array}{c} \rho w\\ \rho wu\\ \rho wv\\ \rho w^{2}+p \end{array}] \end{array}$$ (2)

In order to discretize Eq 1 by using the SPH-ALE, the Riemann problem for two interacting particles *i* and *j* becomes:

$$\frac{\partial \Phi }{\partial t}+\frac{\partial }{\partial x}(F_{E}\left(\Phi \right)\cdot n_{ij}-u_{0}\left(x_{ij},t\right))\cdot n_{ij}\Phi )=0,$$ (3)

where $\Phi \left(x^{n_{ij}},0\right)=\{\begin{array}{c} \Phi_{i},\;if\;x^{\left(n_{ij}\right)}<0\\ \Phi_{j},\;if\;x^{\left(n_{ij}\right)}>0 \end{array}$

where **Φ**_*i*_ and **Φ**_*j*_ are the states of particles *i* and *j* respectively, **n**_*ij*_ is the unit vector with direction from particle i to particle j, *x*_*ij*_ is the midpoint between the two particles and $x^{n_{ij}}$ is the x-coordinate at the local coordinate system. Based on Vila [41], the 3D Euler equations for the particle *i* in the SPH-ALE formulation become:

$$\frac{d}{dt}\left(r_{i}\right)=u_{0,i},$$ (4)

$$\frac{d}{dt}\left(\omega_{i}\right)=\omega_{i}\sum_{j=1}^{N}\omega_{j}\left(u_{0,j}-u_{0,i}\right)\cdot \nabla_{i}W_{ij},$$ (5)

$$\frac{d}{dt}\left(\omega_{i}\Phi_{i}\right)+\omega_{i}\sum_{j=1}^{N}\omega_{j}2G_{E}\left(\Phi_{i},\Phi_{i}\right)\cdot \nabla_{i}W_{ij},$$ (6)

where **G**_*E*_ is given by:

$$\begin{array}{c} G_{E}^{1}=[\begin{array}{c} \rho_{E}\left(u_{E}-u_{0}\right)\\ \rho_{E}u_{E}\left(u_{E}-u_{0}\right)+p_{E}\\ \rho_{E}u_{E}\left(v_{E}-v_{0}\right)\\ \rho_{E}u_{E}\left(w_{E}-w_{0}\right) \end{array}],\;G_{E}^{2}=[\begin{array}{c} \rho_{E}\left(v_{E}-v_{0}\right)\\ \rho_{E}v_{E}\left(u_{E}-u_{0}\right)\\ \rho_{E}v_{E}\left(v_{E}-v_{0}\right)+p_{E}\\ \rho_{E}v_{E}\left(w_{E}-w_{0}\right) \end{array}] \end{array}$$ (7)

$$\begin{array}{c} G_{E}^{3}=[\begin{array}{c} \rho_{E}\left(w_{E}-w_{0}\right)\\ \rho_{E}w_{E}\left(u_{E}-u_{0}\right)\\ \rho_{E}w_{E}\left(v_{E}-v_{0}\right)\\ \rho_{E}w_{E}\left(w_{E}-w_{0}\right)+p_{E} \end{array}] \end{array}$$ (8)

where *ω*_*i*_ refers to the volume of the particle *i* and the *E* subscript indicates the solution from the Riemann problem between the particles *i* and *j*. The pressure is calculated from the Tait equation of state:

$$p_{i}=k\rho_{i}^{n}-B,$$ (9)

where $B=\rho_{0}c_{0}^{2}/n=4.36\;GPa$ is the stiffness parameter and $k=B/\rho_{0}^{n}$, *ρ*_0_ = 7300.0 *kg*/*m*^3^ is the reference density of tin [17], *n* = 7.15 is a parameter (see [55]) and *c*_0_ is the speed of sound, *c*_0_ = 2000 *m*/*s* [17]. In general, Tait equation is found to be a very good approximation to the behaviour of liquids at a wide variety of temperatures and pressures [56] and, thus it is used in the present study to model the behaviour of molten tin droplet.

The whole phenomenon takes place in high vacuum; the ambient pressure is less than 10^−4^ *mbar* which gives an air density of ∼ 10^−6^ *kg*/*m*^3^. Tin saturation pressure at the temperature of the experiment (140*C*) is practically zero (10^−27^ *Pa*), so the vapour density is also practically zero. Consequently, the phase change can be omitted and the process resembles fracture. In the actual experiment, the high intensity laser pulse generates immense localized heating and plasma formation. Temperatures locally may reach 10^7^ *K* and pressures in the order of Mbar [23]. Such transitions have not been modelled in the present simulation due to the immense complexity of modelling dense plasma (hence the approximation of using the Tait equation). Moreover, the heat diffusion time scale is much larger than the duration of the phenomenon [23], thus heat transfer is omitted. In essence, the phenomenon is inertia dominated in the interval examined, justifying the use of the selected models.

First order (Godunov method) and second order (MUSCL-Hancock [57]) spatial accuracy schemes have been implemented in the algorithm (see [53, 54]).

The theoretical value of the tensile strength for Sn is *p*_*theor*_ = −6.8 *GPa* [58], whereas the value of tensile stress varies a lot between experiments and may range from −1.9*GPa* to −0.1*GPa* [17, 59]. Apart from being difficult to measure liquid tension, it can be many times smaller than the theoretical value [60], due to impurities. In practice, a cut-off pressure of *p*_*cut*−*off*_ = −50 *MPa* was selected [61] for the simulations in order to take into account non-ideal effects which induce nucleation of cavitation (e.g. contaminants) [60]. A parametric study was performed regarding the value of the cut-off pressure and its effect on the formation or not of the hollow structure in the middle of the liquid metal droplet. The cut-off values for which simulations have been performed are: *p*_*cut*−*off*_ = {−1.9*GPa*, −1*GPa*, −0.1*GPa*, −0.05*GPa*, −0.01*GPa*, −0.001*GPa*}. The selected (or smaller magnitude) cut-off pressure of −0.05*GPa* allowed the formation of the hollow structure. Larger magnitude cut-off pressures did not allow a cavity to form. Similar observations have been noticed by Basko et al. [25]. Agreement with the experiments was observed when a critical tin pressure of 52 *MPa* was utilized [61] in their simulations. The spherical droplet was transformed to a shell containing two cavities inside, separated by a thin wall. However, choosing a critical pressure of 250 *MPa*, as suggested by Ternovoi et al. [62], led to no such resemblance and the formation of the two lobes could not be modelled.

## 3 Results

In S1 and S2 Appendices the solver is validated for the Riemann problem and for the implosion case respectively, at pressure conditions similar to the liquid metal droplet simulation. The Riemann problem was selected to confirm that the correct wave pattern can be predicted, whereas the implosion case was invoked to validate the algorithm in more than one dimensions.

**Fig 1:**
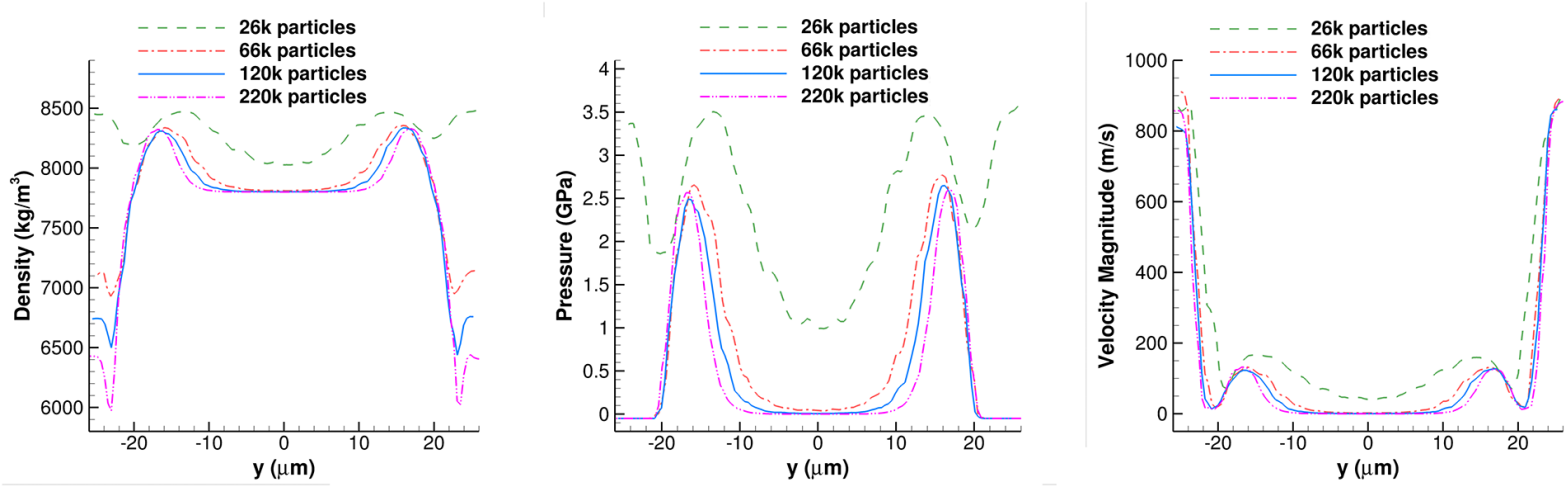
Particle independence study for the liquid metal droplet. Plots of density (left), pressure (middle) and velocity magnitude (right) along a line parallel to the y-axis (*x* = 0 and *z* = 8 *μm*) at *time* = 9 *ns*.

**Fig 2:**
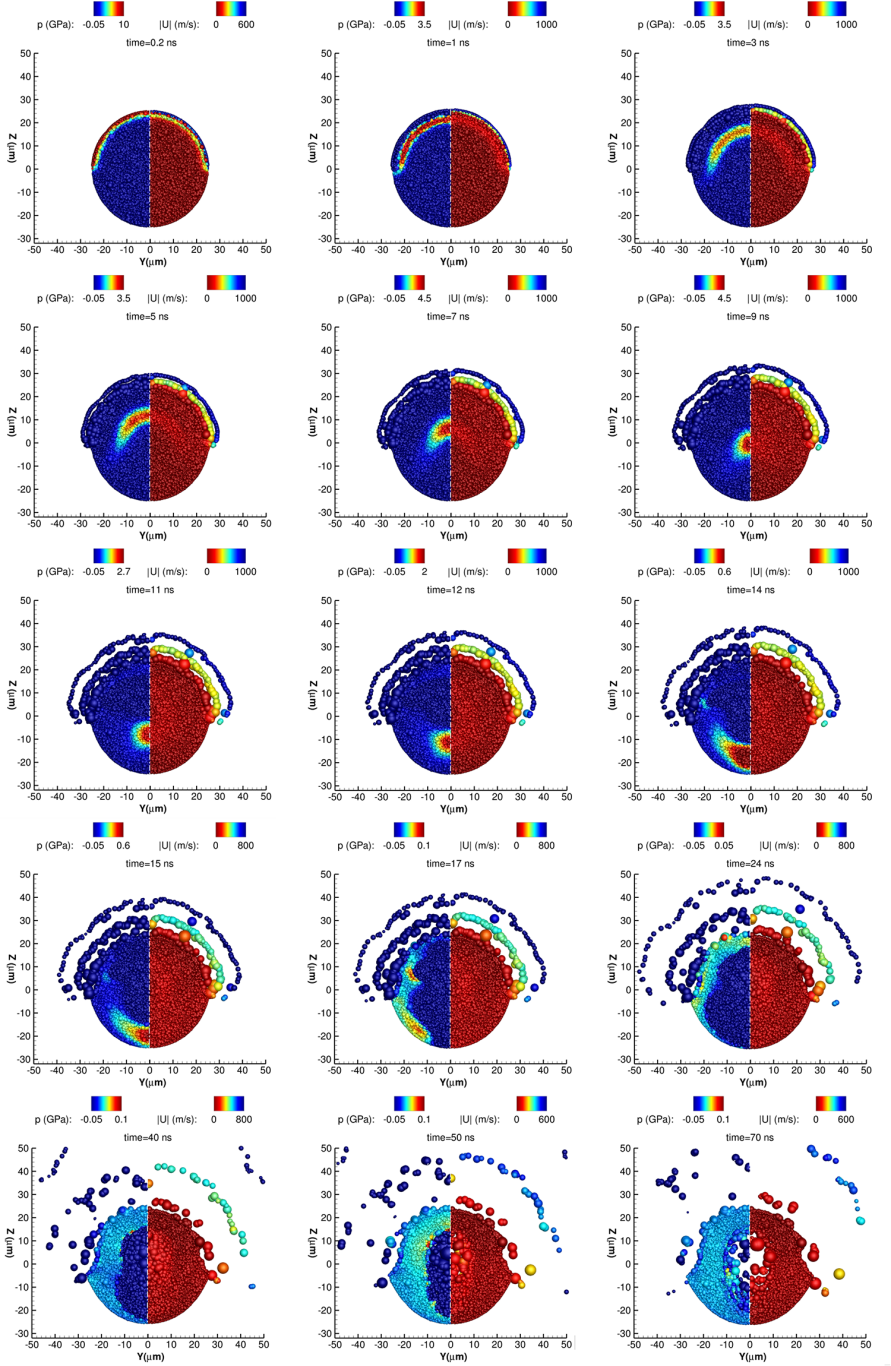
Representation of a thin slice of the sphere to demonstrate the formation of the laser induced cavity. A thin slice of the sphere (*x* ∈ [−1, 1] *μm*) is depicted in the above figures. Pressure (left, negative of the y-axis) and velocity magnitude (right, positive of the y-axis) fields. The size of the particles is visualised based on their volume.

A three dimensional liquid metal (Sn-In) droplet of diameter *D* = 50 *μm* is simulated with the above described SPH-ALE algorithm. The initial location of the particles is given by the cell centers of a pseudo-grid. An unstructured pseudo-grid consisting of tetrahedral cells is created inside the liquid sphere, by defining the number of the discretization nodes on the radius and on the quarter circle arcs. In order to model the laser pulse which impacts the liquid metal droplet in [26], a constant pressure field of *p*_*init*_ = 1.25 ⋅ 10^11^ *Pa*, based on the ablation pressure scaling law [63], is applied as initial condition on the outer shell of the hemisphere with positive z-axis values (the corresponding density from Tait EoS is *ρ*_*init*_ = 12530.0 *kg*/*m*^3^), while the pressure in the rest of the droplet is zero (corresponds to density of *ρ*_0_ = 7300.0 *kg*/*m*^3^). The thickness of the outer shell is actually the initial diameter of the droplets and thus, it depends on the particles population. Nevertheless, particle independence study (see Fig 1) showed that after a point, the pressure distribution inside the droplet is not affected by the particles resolution. The solution after 1 time step is shown in Fig 2 at *time* = 0.2 *ns*, which is a good estimation of the initial set up.

A particle independence study is performed in order to evaluate the number of the particles which will accurately model the phenomenon. Four different particle populations have been employed, 25*k* particles (coarser case), 66*k* particles (coarse case), 120*k* particles (fine case) and 200*k* particles (finer case). In Table 1 all the cases simulated during the grid independence study are summarised and in Fig 1 the density (left), pressure (middle) and velocity magnitude (right) along the *y* − *axis* are plotted. The finer grid has been selected in order to accurately model the phenomenon, although similar results have been derived by using 66*k* particles and above. It is evident that for a simulation with higher population of particles, a symmetrical profile is acquired, which was not the case for the low population simulations, where asymmetries along the the *y* − *axis* were noticed. Although the initial condition (*p*_*init*_) depends on the particles population, as in a coarser simulation the particles size and the shell thickness would be larger, particle independence study showed that the above initialization doesn’t affect the pressure distribution at later times.

**Table 1:** Case names, discretization of the sphere radius and of the quarter circle arc and the total number of the particles used in the sphere.

Name	discretization (intervals in radius)	number of particles
*coarser*	15	25001
*coarse*	20	66084
*fine*	25	119428
*finer*	30	220772

**Fig 3:**
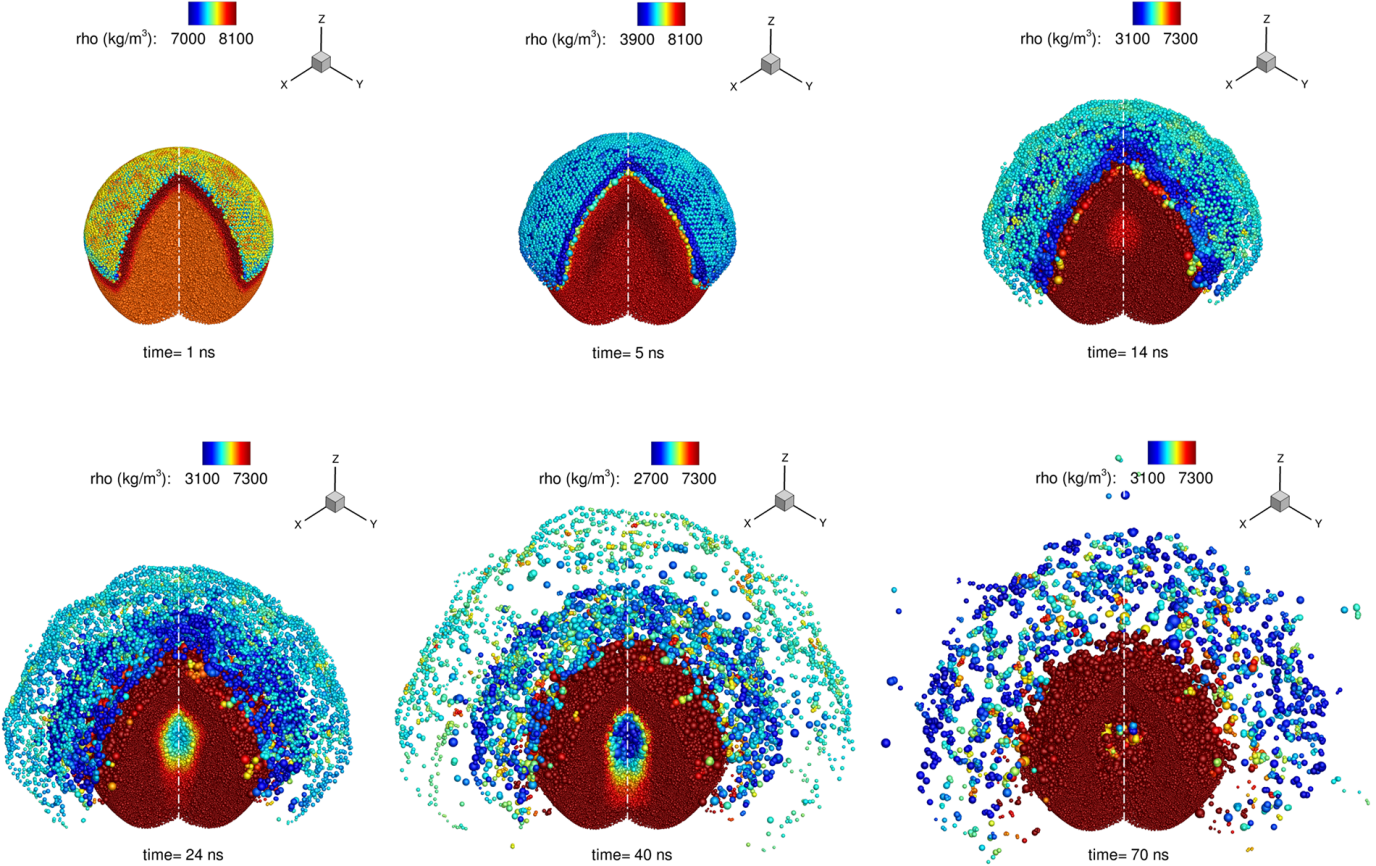
Three dimensional representation of the density field in the liquid metal droplet. The hemispherical shell at the top of the droplet expands forming an ablation pattern (*time* = 1 − 14 *ns*). At the same time interval the shock wave propagates in the interior of the droplet, converging in the center. Afterwards, vacuum is formed in the center of the droplet, as indicated with the low density (blue colour in the figure, *time* = 40 *ns*) and the particles become separated forming a hollow cavity.

In Fig 3 a 3-D view of the liquid metal droplet is shown; the particles are coloured by the density field and the ablation pattern is demonstrated. A similar pattern was predicted in the 2-D numerical simulations of Basko et al. [25] when they used approximately the same critical pressure (52 *MPa*) as in the present study. Because of the pressure wave, the density is initially increased in the upper hemispherical shell. At later times the upper hemispherical part of the droplet expands and the ablation pattern is evident, while a density decrease in the center of the droplet is noticed at *time* = 40, 70 *ns*, which is actually due to the creation of a hollow structure, as it is shown more clearly in Fig 2.

In Fig 2 the particles population on a thin slice (*x* ∈ [−1, 1] *μm*) is shown for several time frames in order to demonstrate the wave dynamics and the cavity which is created in the center of the droplet. The particles are coloured by the pressure (negative *y* − *axis*) and the velocity magnitude (positive *y* − *axis*). The initial pressure and velocity magnitude fields due to the laser pulse are shown at *time* = 0.2 *ns*. At *time* = 1, 3, 5 *ns*, the radial shock waves are moving towards the center of the droplet, due to the deposited *p*_*init*_ on the upper hemispherical layer of the droplet. The superposition of the radial shock waves when they converge, is focusing at the center, which is evident at *time* = 7, 9 *ns*. Similar findings have been also demonstrated by Reijers et al. [8], Klein et al. [30]. As this focused compressed area propagates downwards, it is followed by a rarefaction wave (*time* = 11 *ns* and *time* = 12 *ns*) which creates a pressure drop beyond the tensile strength, resulting in material rupture [23] (first cavity). As soon as the shock wave passes the center of the droplet, it expands (*time* = 11, 12, 14 *ns*). At *time* = 15 *ns* the compressed area has reached the bottom of the droplet interface (most negative *z* − *axis*). Then the compression area is reflected and an expansion wave is created which moves upwards. When the latter meets the first rarefaction wave which is still following the shock wave, a second cavity in the lower hemisphere of the droplet is formed (*time* = 17, 24, 40 *ns*). The superposition of the two rarefaction waves causes an additional pressure decrease which may also lead to potential rupture [23] in the center of the droplet (*time* = 50, 70 *ns*). In the last frame the absence of particles in the center of the droplet is evident, which denotes the existence of a hollow structure. This phenomenon is referred as spallation [23, 64] and optically, 2 lobes of low pressure have been identified both in the present simulation and in similar experimental studies, either in liquid metal droplets which impact a laser pulse [23, 25] or in water droplets subjected to shock waves [15]. The ablation because of the superposition of the radial pressure waves is evident (Figs 3 and 2) and it is starting from *time* = 5 *ns*. The velocity magnitude field is thus increased (up to 1000 *m*/*s*) on the upper hemispheric shell and at later times of Fig 2 the particles have been scattered away from the droplet. Similar ablation patterns have been documented in several experimental works [23, 25, 30]. Only a qualitative comparison can be made between Fig 3 of [25] at *t* = 1 *μs* and Fig 3 of the present work at *t* = 17 *ns*, since the former was purely 2-D.

## 4 Conclusions

A SPH-ALE algorithm for the simulation of laser-induced deformation of a liquid metal droplet has been utilised. Compared to traditional mesh methods, such as Finite Volumes, where the liquid and the gas phases would have been discretised, SPH is more efficient, since only the liquid phase is modelled. Furthermore, the liquid metal deformation is easily handled by the SPH-ALE method without applying any mesh deformation techniques and without the need of an explicit interface treatment. The shock and expansion waves responsible for the ablation and the generation of the two cavities in the center of the droplet have been demonstrated. When the waves interact with the bottom free surface, a hollow cavity is created. The wave dynamics pattern, the ablation and the cavity formation, predicted by the present numerical work, are similar to prior experimental results.

## Supporting information

S1 Video 3-D animation of the laser-droplet interaction. Representation of the laser interaction with the droplet showing the pressure (left) and density (right) fields. A slice has been blanked to show the distribution in the interior of the droplet. The white dashed line denotes the axis of symmetry.
(MPG)

S1 Appendix Riemann problem (Validation). A 1-D shock tube case is examined to assess the accuracy of the method.
(PDF)

S2 Appendix Implosion problem (Validation). A 2-D implosion case is examined to assess the accuracy of the method.
(PDF)

S3 Appendix Surface tension and viscosity effects. Investigation of the viscosity and surface tension effects on the modified Rayleigh-Plesset equation.
(PDF)
