# Supplementary material for: Smoothed particle hydrodynamics simulation of a laser pulse impact onto a liquid metal droplet
Source: PLoS One. 2018 Sep 25;13(9):e0204125. doi: 10.1371/journal.pone.0204125 (PMC6155526; doi:10.1371/journal.pone.0204125)
Supplement: S1 Appendix — A 1-D shock tube case is examined to assess the accuracy of the method. (PDF) [file pone.0204125.s002.pdf]

S1 Appendix: Riemann problem (Validation)

In this section, the Riemann problem is examined in order to validate the accuracy of the numerical scheme. The computational domain is $x \in [-25, 25]\,\mu m$, whereas the initial conditions for the left state ($x \leq 0$) are: $\rho_L = 12750\,kg/m^3$, $p_L = 2160648.25\,bar$, $U_L = 0\,m/s$ and for the right state ($x \geq 0$) are: $\rho_R = 7300\,kg/m^3$, $p_R = 0\,bar$, $U_R = 0\,m/s$. The parameters for the Tait equation (Eq. 9) have been selected as follows: $\rho_0 = 7300\,kg/m^3$, $c_0 = 2000\,m/s$ and $n = 7.15$. Wave transmissive boundary conditions have been used for the left and the right sides. In Fig. 1 comparison between the exact and the SPH solutions, with Eulerian and Lagrangian perspective, is shown at $Time = 1.5\,ns$ by utilising 501 particles and second order of spatial accuracy. The two numerical solutions are in satisfactory agreement with the exact one and the wave pattern has been captured.

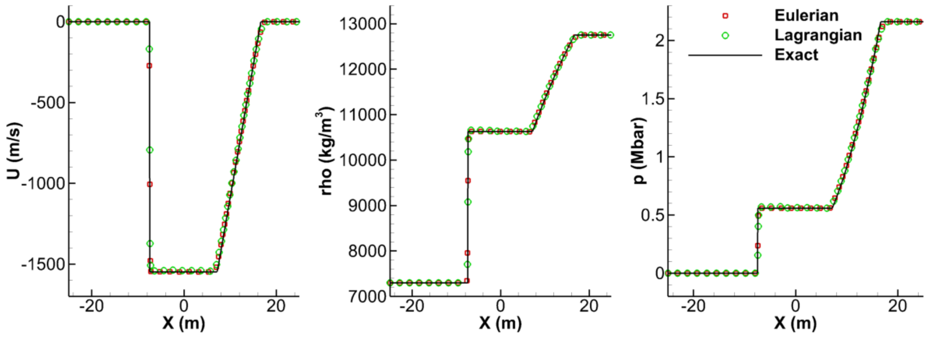

Fig 1. Validation of the SPH solver for the Riemann problem.
Comparison of the x-velocity (left), density (middle) and pressure (right) between the exact and the numerical solution, either with Eulerian or Lagrangian perspective, at $Time = 1.5\,ns$. Second order of spatial accuracy with 501 particles has been used.
