# Supplementary material for: Smoothed particle hydrodynamics simulation of a laser pulse impact onto a liquid metal droplet
Source: PLoS One. 2018 Sep 25;13(9):e0204125. doi: 10.1371/journal.pone.0204125 (PMC6155526; doi:10.1371/journal.pone.0204125)
Supplement: S2 Appendix — A 2-D implosion case is examined to assess the accuracy of the method. (PDF) [file pone.0204125.s003.pdf]

S2 Appendix: Implosion problem (Validation)

The implosion problem is simulated as a validation of the fluxes in the x,y directions. The computational domain is $(-187.5, 187.5) \times (-187.5, 187.5)\,\mu m$ and the initial conditions for distance from the origin $R \leq 20\,\mu m$: $\rho_{in} = 7300\,kg/m^3$, $p_{in} = 0\,Pa$, $U_{in} = 0\,m/s$ and for $R \geq 20\,\mu m$: $\rho_{out} = 12750\,kg/m^3$, $p_{out} = 2160648.25\,bar$, $U_{out} = 0\,m/s$. The parameters for the Tait equation are the same as in S1 Appendix. Wave transmissive boundary conditions have been used for all four sides. In Fig. 1 comparison between the reference FV solution (with discretization of 500 cells) and the SPH-ALE solution is shown at $Time = 5.75\,ns$ for second order of spatial accuracy. The implosion case is similar to the actual experiment since it involves shock wave focusing at the origin. It demonstrates that the solver can track accurately shock waves in the multi-dimensional applications with a much coarser resolution that the reference solution (250×250 particles in total, 27 particles in the diameter of the imploding region compared to 5000 cells of the high order reference FV solution).

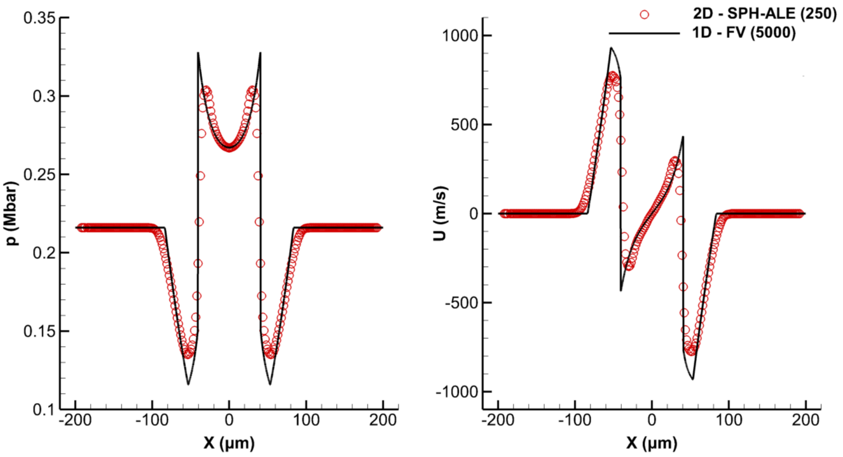

Fig 1. Validation of the SPH solver for the implosion problem. Comparison of pressure (left) and the x-velocity (right) between the 1-D axisymmetric FV solver and the numerical solution acquired by the SPH-ALE method at $Time = 5.75\,ns$. Second order of spatial accuracy with 250 particles in each direction has been used (27 particles in the diameter of the imploding region), whereas for the FV solver, 5000 cells have been utilised as reference.
