# Supplementary material for: Smoothed particle hydrodynamics simulation of a laser pulse impact onto a liquid metal droplet
Source: PLoS One. 2018 Sep 25;13(9):e0204125. doi: 10.1371/journal.pone.0204125 (PMC6155526; doi:10.1371/journal.pone.0204125)
Supplement: S3 Appendix — Investigation of the viscosity and surface tension effects on the modified Rayleigh-Plesset equation. (PDF) [file pone.0204125.s004.pdf]

S3 Appendix: Surface tension and viscosity effects

In the present study, the viscous and the surface tension effects were not considered, since their contribution during the simulated interval is negligible. In order to prove this point, the modified Rayleigh-Plesset equation is considered [26], which is describing the rate change of a bubble radius R_b inside a droplet of radius R_d:

$$(1-\lambda)R_b\ddot{R}_b + \left(\frac{3}{2} - 2\lambda + \frac{1}{2}\lambda^4\right)\dot{R}_b^2 = \frac{p_0 R_{b0}^{3\gamma}}{R_b^{3\gamma}\rho_l} - \frac{2\sigma}{\rho_l R_b} - \frac{2\sigma}{\rho_l R_d} - \frac{4\mu\dot{R}_b}{R_b\rho_l}, \tag{10}$$

where $\lambda = R_b/R_d$ and the droplet radius is found from mass conservation and assuming constant density $R_d = \left(R_{d0}^3 - R_{b0}^3 + R_b^3\right)^{1/3}$ with $R_{b0} = 5\,\mu m$ and $R_{d0} = 25\,\mu m$ being the initial radius of the bubble and the droplet respectively. The rest of the parameters are as follows: $p_0 = 900\,bar$, $\gamma = 1.4$, $\rho_l = 7300\,kg/m^3$, $\sigma = 0.534\,N/m$ and $\mu = 0.00175\,Pas$, aiming to replicate the case from [26]. From the solution of the above differential equation, the effect of the viscosity and the surface tension is negligible for times $< 1.5\mu s$, as it can be seen in Fig. 1. In addition, the Laplace pressure is $\Delta p = \frac{2\sigma}{R} = \frac{2\cdot 0.534\,N/m}{25\cdot 10^{-6}\,m} \approx 0.4\,bar$ and it is insignificant in respect to the e.g. $1.25\,Mbar$, or the tension level of $-500\,bar$. Given the fact that the main focus here is the violent dynamics of nucleation (shock wave focusing, rarefaction wave, liquid tension) and thus the final simulation time is $\sim 0.1\mu s$, it is safe to omit surface tension and perform inviscid simulations. To give quantitative data, at $0.1\,\mu s$ the maximum deviation at bubble radius when including or omitting surface tension/viscosity is 0.17%. At $1\,\mu s$ the maximum deviation grows up to 2.1%. However, surface tension will strongly affect the phenomenon at later times ($> 4\,\mu s$) of the droplet-bubble evolution, since it is the only mechanism of pressure formation inside the droplet (Laplace pressure). This means omission of surface tension would lead to unconstrained expansion of the droplet, at least for the 1-D case, as modelled with the modified Rayleigh-Plesset equation.

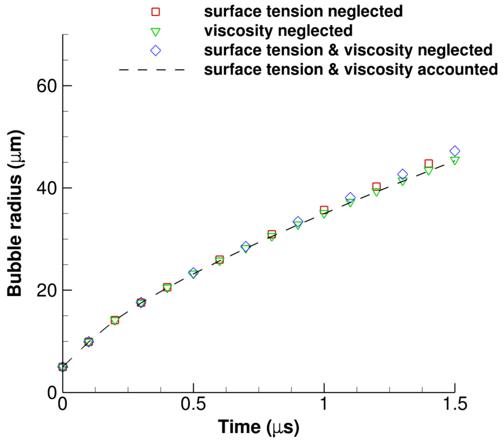

Fig 1. The impact of the surface tension and viscosity on the bubble radius change with respect to time.
Four different configurations have been tested: surface tension is neglected (square), viscosity is neglected (gradient), surface tension and viscosity are neglected (diamond) and surface tension, viscosity are both taken into account (dashed line).
